# Silver Nanoparticles as Antifungal Agents in Acrylic Latexes: Influence of the Initiator Type on Nanoparticle Incorporation and *Aureobasidium pullulans* Resistance

**DOI:** 10.3390/polym15061586

**Published:** 2023-03-22

**Authors:** Gabrielle Boivin, Anna M. Ritcey, Véronic Landry

**Affiliations:** 1 Département de Chimie, Université Laval, 1045 Avenue de la Médecine, Québec City, QC G1V 0A6, Canada; 2 FPInnovations, 1055 rue du PEPS, Québec City, QC G1V 4C72, Canada; 3 Département des Sciences du Bois et de la Forêt, Université Laval, 2425 rue de la Terrasse, Québec City, QC G1V 0A6, Canada

**Keywords:** silver nanoparticles, antifungal properties, acrylic polymers, miniemulsion polymerization, coatings, wood protection

## Abstract

Discoloration of wood coatings due to fungal growth negatively affects the aesthetic properties of the coatings, and new ways to control fungal growth on coatings are needed. For this reason, silver nanoparticles (AgNPs) have been incorporated in acrylic latexes as antifungal agents. Using miniemulsion polymerization, latexes were prepared with two types of initiators (hydrophilic and hydrophobic) to assess the influence of the initiator type on AgNPs dispersion, both within the latex particles and the dry film. In addition, the impact of NP dispersion on resistance to black-stain fungi (*Aureobasidium pullulans*) was also evaluated. Inductively coupled plasma optical emission spectroscopy (ICP-OES) analysis indicates that acrylic latexes prepared with azobisisobutyronitrile (AIBN) as the initiator contain more AgNPs than those prepared with potassium persulfate (KPS). Cryo-TEM and SEM analyses show that the distribution of the AgNPs within the polymer particles is influenced by the nature of the initiator. When AIBN, a hydrophobic initiator, is used, the AgNPs appear to be closer to the surface of the polymer particles and more evenly distributed. However, the antifungal efficiency of the AgNPs-embedded latexes against *A. pullulans* is found to be higher when KPS is used, despite this initiator leading to a smaller amount of incorporated AgNPs and a less uniform dispersion of the nanoparticles.

## 1. Introduction

Translucent wood coatings in exterior applications require frequent refinishing and maintenance due to discoloration caused by fungal growth and photodegradation. The fungi responsible for wood discoloration are commonly referred to, in the literature, as staining fungi or black-stain fungi. These fungi blacken wood by metabolizing photodegradation by-products into melanin. This discoloration is perceived as problematic by consumers as it alters the aesthetic properties of wood products, which reduces their service life. The most important strains of black-stain fungi are the ascomycetes *Aureobasidium pullulans*, *Eppicocum nigrum* and *Sclerophoma pithyophila* [1,2]. *A. pullulans* is considered one of the toughest strains to control [3].

Typically, fungicides are used to extend the service life of such coatings. However, these additives can be vulnerable to photodegradation, volatilization, and leaching, which reduce the coating’s long-term efficacy. When compared with organic biocides and fungicides, metallic nanoparticles have numerous advantages, such as good stability at high temperatures, resistance to degradation, and low volatility. Moreover, the incorporation of nanomaterials such as zinc oxide (ZnO) [4,5], cerium oxide (CeO_2_) [6], copper oxide (CuO) [7], titanium oxide (TiO_2_) [8], and silver (Ag) [3] can provide antibacterial and antifungal properties to wood coatings. Nevertheless, silver is considered one of the most efficient antibacterial agents [9,10].

In recent years, AgNPs have proven their antimicrobial efficacy in wood coatings in several studies [11,12,13,14]. Other studies address the antifungal efficacy of AgNPs of different sizes, forms, and concentrations, in wood applications [3,15,16,17]. Except for our previous work [3], all studies reported the post-addition of AgNPs to a binder or carrier through simple mixing rather than the encapsulation of NPs during the polymerization stage. Moreover, previously cited studies on the incorporation of AgNPs in wood coatings did not investigate the impact of AgNP dispersion and localization in the dry film on their fungal resistance. Since the antifungal efficacy of AgNPs is influenced by the quality of their dispersion within the polymer particles and nanoparticle aggregation can result in a decrease in antimicrobial and antifungal properties [18], the study of these parameters is important. In the present work, miniemulsion polymerization is employed to improve nanoparticle dispersion in the polymer matrix through particle encapsulation [19]. Two different types of initiators (oil-soluble and water-soluble) are used in the synthesis of acrylic latexes to assess their impact on AgNP incorporation and dispersion.

Both oil-soluble and water-soluble initiators have been used in miniemulsion polymerization, although the latter is the more frequently employed [20]. Oil-soluble initiators such as azobisisobutyronitrile (AIBN) are considered less effective since they decompose within the monomer droplets and can then recombine with each other and become inactive. On the other hand, water-soluble initiators such as potassium persulfate (KPS) decompose in the aqueous phase and can then form oligomeric radicals through reaction with the monomers present in the aqueous phase before entering the monomer droplets. Chern and Liou [21] compared the effect of the initiator (AIBN vs. KPS) on nucleation. It was reported that the oil-soluble initiators promote nucleation in the monomer droplets, whereas the water-soluble initiators favor homogenous/micellar nucleation. These observations suggest that the type of initiator will influence the morphology of polymer-inorganic nanocomposites prepared by this technique. In turn, differences in nanoparticle distribution will affect the antifungal properties of AgNPs-embedded acrylic latexes. Thus, the purpose of this work is to evaluate the influence of the type of initiator on the antifungal properties of AgNPs-embedded in acrylic latexes. The amount of silver present in synthesized latexes with KPS and AIBN is determined by inductively coupled plasma optical emission spectroscopy (ICP-OES). The dispersion of AgNPs in acrylic films is also investigated with SEM. Finally, the resistance to *A. pullulans* of the acrylic latexes prepared is evaluated.

## 2. Materials and Methods

### 2.1. Materials

Methyl methacrylate (MMA), butyl acrylate (BuA), and acrylic acid (AA) were obtained from Sigma-Aldrich (Oakville, ON, Canada) and purified on a basic aluminium oxide column before use, to remove the inhibitor. Silver nitrate (AgNO_3_), oleylamine, hexadecane (HD), sodium dodecylsulfate (SDS, 99%), azobisisobutyronitrile (AIBN, 98%), and potassium persulfate (KPS, 99%), also from Sigma-Aldrich, were used as received. Polyphase 100 (containing 98% of iodopropynyl butylcarbamate (IPBC)) from Troy Corp. (Florham Park, NJ, USA), Wocosen Technical (propiconazole) from Janssen (Raritan, NJ, USA), AMP-95 from Angus Chemical (Buffalo Grove, IL, USA), and the acrylic resin, Acronal 4110, from BASF (Mississauga, ON, Canada), were all used as received.

### 2.2. Silver Nanoparticles Synthesis

A previously reported method from Chen et al. [22] was used to prepare 10–15 nm colloidal AgNPs. Details of the synthesis are provided elsewhere [3]. Silver nitrate (AgNO_3_) was dissolved in oleylamine and paraffin, and mechanically stirred under nitrogen for 20 min at room temperature. Furthermore, the solution was heated to 180 °C using a rate of 4 °C/min. To ensure silver reduction, the temperature was maintained at 180 °C for 2 h. The temperature was then decreased to 150 °C and maintained for 6 h. Once the solution had cooled to room temperature, chloroform and acetone were added and the solution was centrifugated to wash the NPs.

### 2.3. Latex Synthesis and Formulation Preparation

Miniemulsion polymerization was used to prepare latex polymer particles following a procedure described elsewhere [3]. First, monomers and hexadecane were mixed in a beaker. In a second beaker, the aqueous phase was prepared by dissolving SDS in ultrapure water (Nanopure^®^ water system (Thermo Fisher Scientific; Waltham, MA, USA)). The organic phase was mixed with the aqueous phase and stirred mechanically for 1 h. Afterwards, the mixture was sonicated using an ultrasonic probe (Model 500 Dismembrator; Thermo Fisher Scientific; Waltham, MA, USA). In parallel, the initiator (KPS) was dissolved in ultrapure water. The initiator solution was then added to the emulsion and mixed in a three-neck round-bottom flask, and purged under nitrogen for 20 min. The system was heated under reflux for 3 h at 70 °C. When AIBN was used, the same protocol was followed except that 1% (with respect to monomer weight) was dispersed in the organic phase and the system was heated at 78 °C rather than 70 °C.

Latexes containing AgNPs were prepared using the same protocol described above. The only difference was that 1% (with respect to monomer weight) of AgNPs were added to the organic phase before sonication. The prepared latexes were mixed in a ratio of 1:1 (wt/wt) with a commercial acrylic resin (Acronal 4110) to increase the formulation solid content to 30%. A previous study showed that a combination of IPBC and propiconazole was able to control the growth of *A. pullulans* and *E. nigrum* [23]. Based on these observations, a latex was also prepared with 0.1 wt% of Polyphase 100 (98% IPBC) and 1 wt% of Wocosen Technical (propiconazole) as a reference. All formulations prepared contain 1 wt% Tinuvin 292 and 1 wt% Tinuvin 1130 as UV absorbers and HALS, respectively. The prepared latexes and their components are shown in Table 1.

### 2.4. Material Preparation

#### 2.4.1. Free-Standing Film Preparation

Latexes containing AgNPs polymerized with KPS and AIBN were poured into Teflon molds (50 mm × 50 mm × 1 mm) and were air dried. Once dried, the films were pealed from the molds and used as is for the scanning electron microscopy analysis.

#### 2.4.2. Sample Preparation

White spruce (*Picea glaucea* (Moench) Voss) boards were cut into 57 × 57 × 6 mm samples. The samples were lightly sanded with 80-grit sandpaper and conditioned for 2 weeks at 20 °C/65% RH. Two coats of 4 mils (101 µm) of each formulation (A to D) were applied with a foam brush on 12 samples and allowed to dry for 2 weeks at ambient temperature.

### 2.5. Characterization Methods

#### 2.5.1. Inductively Coupled Plasma Optical Emission Spectroscopy (ICP-OES)

The amount of silver present in synthesized latexes was determined using inductively coupled plasma optical emission spectroscopy (ICP-OES). A mixture of concentrated nitric and sulfuric acids (8:3) was used to achieve dissolution of AgNPs and latex particles. All digestions were conducted using a microwave digestion system MDS-81D by CEM (Matthews, NC, USA). The diluted digestates were then analyzed by ICP-OES with a model 5110 instrument from Agilent Technologies (Santa Clara, CA, USA).

#### 2.5.2. Cryo-Transmission Electron Microscopy (Cryo-TEM)

Latexes containing AgNPs polymerized with KPS and with AIBN were vitrified on TEM grids using a Vitrobot Mark IV System from Thermo Fisher Scientific (Waltham, MA, USA). TEM grids were then transferred into the TEM using a liquid nitrogen cryo-transfer holder Gatan 626 from Ametek (Pleasanton, CA, USA). Observations were made under cold conditions using a Philips CM120 transmission microscope at a voltage of 120 kV.

#### 2.5.3. Scanning Electron Microscopy (SEM)

The dispersion of AgNPs in acrylic films was examined using a scanning electron microscope (SEM). A Inspect model F50 SEM from FEI (Hillsboro, OR, USA) equipped with an Octane Super-A (Edax Ametek) (Warrendale, PA, USA) energy dispersive spectroscopy (EDS) detector was used to evaluate the homogeneity of the dispersion of AgNPs within the acrylic films. Free-standing acrylic films of 1 cm × 1 cm were used for this technique.

#### 2.5.4. Sample Pre-Weathering

White spruce samples coated with the various formulations (A to D) were pre-weathered to initiate photodegradation and generate fungi nutrients (by-products of photodegradation) before being tested against *Aureobasidium pullulans* to accelerate fungal growth. Accelerated UV exposure was conducted over a period of 180 h on three samples per series. Samples were placed in a Q-Sun from Q-Lab (Westlake, OH, USA) in accordance with a modified version of the standard ASTM G155-21 Standard Practice for Operating Xenon Arc Light Apparatus for Exposure of non-metallic substrates [24]. Cycle 1 of the standard method was modified to remove the water spray step to avoid leaching of the by-products of photodegradation. Removing this step increases the nutrients available to support fungal growth. The specific parameters used for the pre-weathering are presented in Table 2.

#### 2.5.5. Black-Stain Fungi Resistance Tests

Black-stain resistance testing procedures are based in part on the ASTM D5590-10 standard [25] and the Nordtest NT Build 338 standard [26], as described by Stirling et al. [23]. First, wood samples were sterilized by irradiation using an ion beam with two passes at 17 kGy (Iotron Industries; Port Coquitlam, BC, Canada). *Aureobasidium pullulans* were selected from FPInnovations’ culture collection from wood coatings affected by black stain in western and eastern Canada (mixture of *Aureobasidium pullulans*-like isolates representing several colony types) and grown on plates of 1% malt extract agar. After two weeks of incubation, a sterile 0.01% surfactant solution (Tween^®^ 80) was poured onto each plate, and the fungi were gently removed from the agar surface. The resulting solution was diluted and blended with a Waring Commercial Blender (Waring; Torrington, WY, USA). The inoculum was then filtered and checked for the presence of spores and mycelia using optical microscopy. The inoculum was sprayed on the surface with an IWATA Eclipse HP-BCS airbrush (ANEST Iwata-Medea, Inc.; Portland, OR, USA). The inoculated samples were then placed in sterile, pre-wetted chambers and incubated at 23.5 °C for four weeks. Each week, they were lightly sprayed. Black stain growth was evaluated every two weeks up to eight weeks and was rated on a scale of 0 to 5, as described in Table 3.

## 3. Results

### 3.1. Inductively Coupled Plasma Optical Emission Spectroscopy (ICP-OES)

ICP-OES analysis was used to determine the concentration of silver in dry acrylic films prepared from formulations containing AgNPs. The results are presented in Table 4. For this study, the same quantity of AgNPs was added initially to the monomer phase before polymerization to achieve a targeted concentration in the final dry film of 1% of total solid weight. ICP-OES analysis indicates that not all the added AgNPs are incorporated in the latexes. Indeed, complete AgNPs incorporation would have led to an Ag concentration of 10 mg/g. Furthermore, ICP-OES confirms that there are approximately twice as many AgNPs embedded in the final dry film when AIBN is employed as the initiator rather than KPS.

### 3.2. Cryo-Transmission Electron Microscopy (Cryo-TEM)

**Figure 1:**
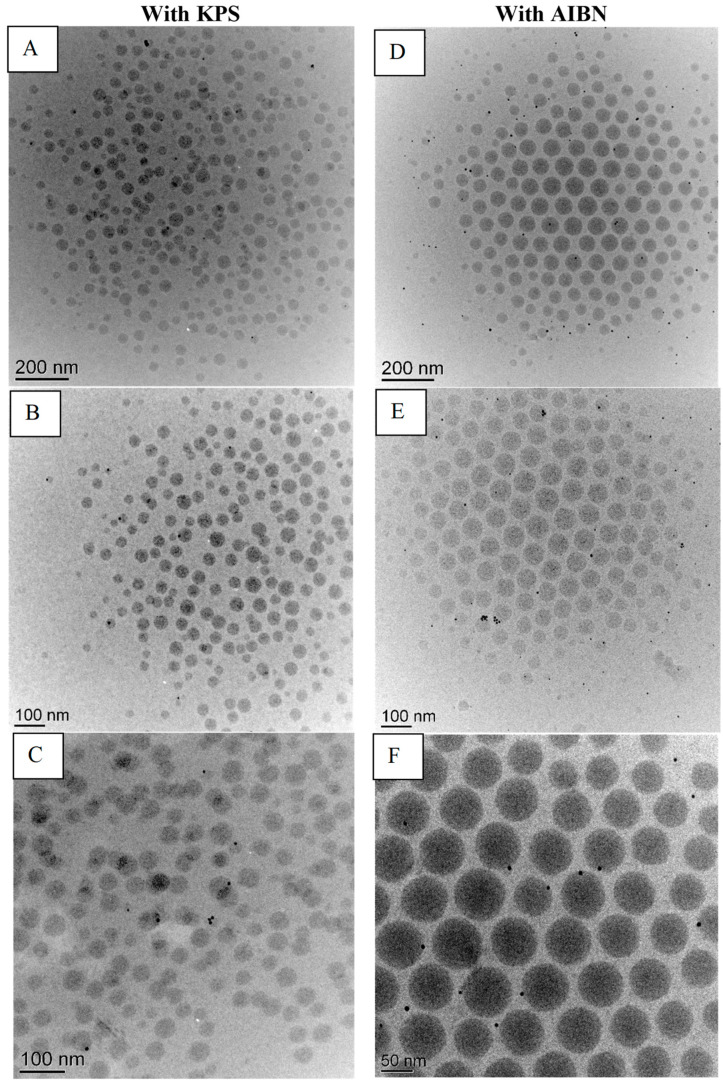
Cryo-TEM micrographs of latexes containing AgNPs prepared with KPS (**A**–**C**) or AIBN (**D**–**F**).

Cryo-TEM images of latexes containing AgNPs prepared using KPS and AIBN, respectively, as the initiators are presented in Figure 1. Both prepared latexes show encapsulated AgNPs in the polymer particles as well as a few free nanoparticles. The differences between the two initiators appear to be related to the localization of the nanoparticles and their dispersion. Specifically, the AgNPs appear to be closer to the surface of the acrylate particles and more evenly distributed when AIBN is used to initiate polymerization. Furthermore, the AgNPs seem to be more evenly distributed when using the hydrophobic initiator compared with the hydrophilic initiator, for which more NP clusters are observed. Moreover, the AIBN prepared latex shows relatively more monodisperse polymer particles than KPS-prepared latexes as well as less AgNPs present.

### 3.3. Scanning Electron Microscopy (SEM)

**Figure 2:**
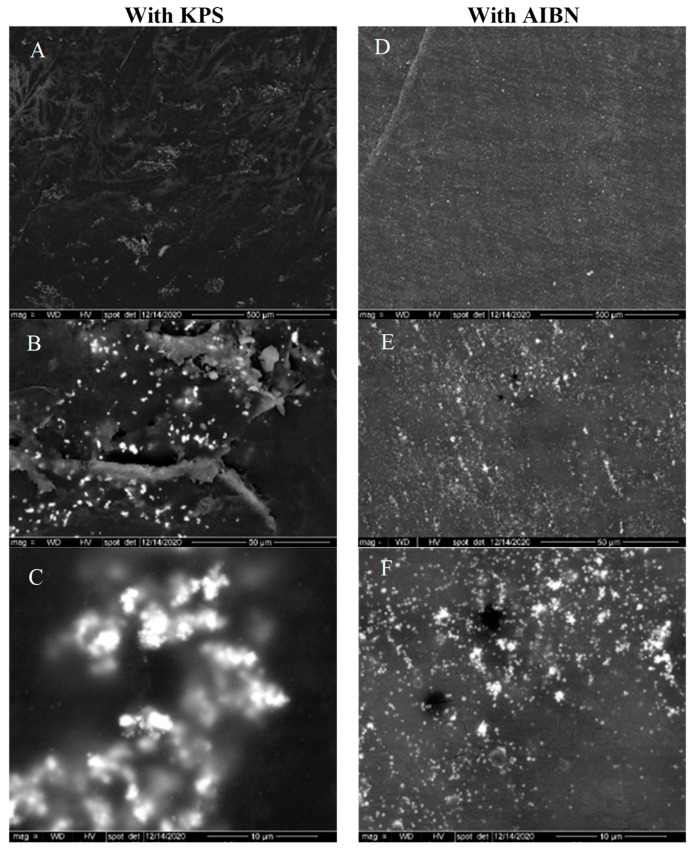
SEM’s pictures of free films containing 1% of AgNPs prepared using KPS (**A**–**C**) or AIBN (**D**–**F**) as initiator.

**Figure 3:**
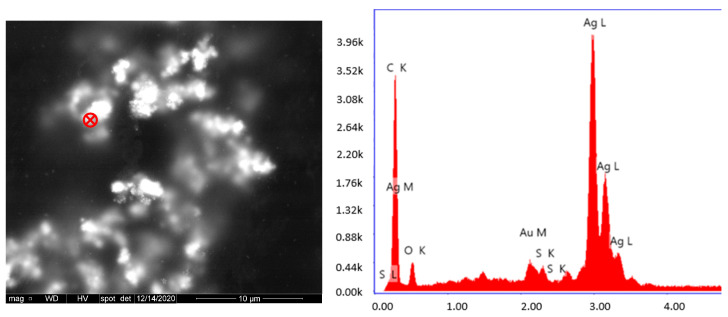
SEM image of acrylic latex containing AgNPs (**left**) and energy dispersive spectroscopy spectrum of the red marker (**right**).

The dispersion of AgNPs in acrylic films was also observed using SEM. Figure 2 shows the SEM images of the top surface of a free-standing acrylic film containing AgNPs. The white spots in the SEM images correspond to AgNPs, as confirmed by the energy dispersive spectroscopy (EDS) analysis presented in Figure 3. According to SEM analysis, the AgNPs encapsulated using the oil-soluble initiator present a relatively uniform dispersion in the free film. On the other hand, AgNPs encapsulated with the water-soluble initiator tend to form larger aggregates as seen in the micrographs of Figure 3. This is probably explained by the less uniform dispersion of AgNPs in the latex, when using KPS (as seen with the cryo-TEM analysis on Figure 1), which is amplified following the coalescence of the polymer particles during film formation.

### 3.4. Black-Stain Fungi Resistance Tests

**Figure 4:**
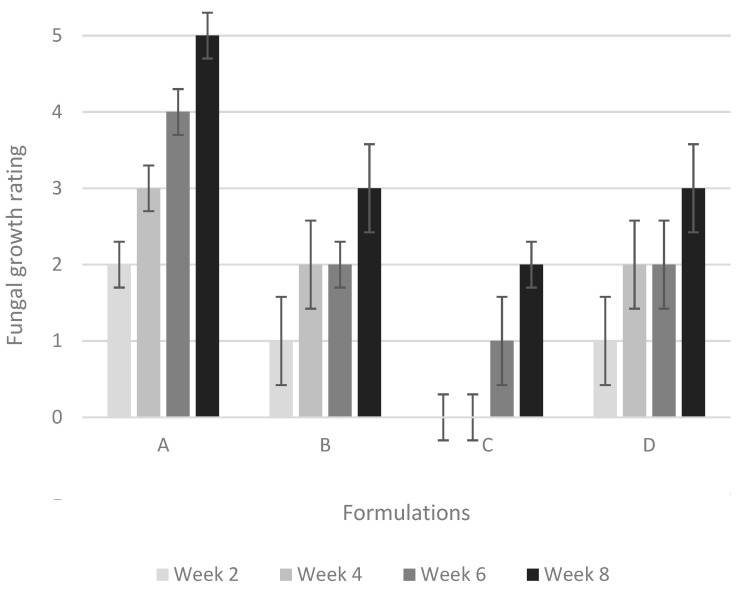
Fungal growth evolution over an eight-week period for samples coated with four different formulations defined in Table 1.

**Figure 5:**
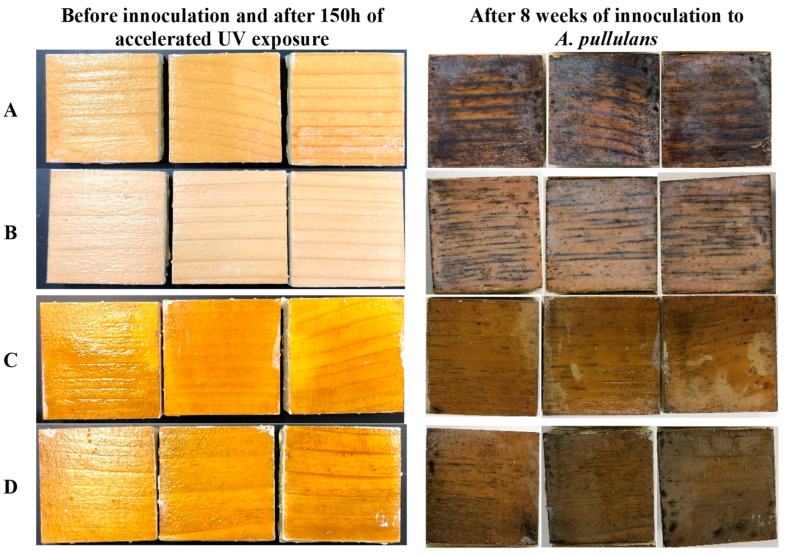
Photographs of samples coated with four different formulations defined in Table 1 after 150 h of accelerated UV exposure, and before and after eight weeks of incubation with *A. pullulans*.

In this work, black-stain testing was performed to assess the ability of the prepared formulations to resist colonization by black-stain fungi. This test is an accelerated aggressive test in which four series of coated samples were inoculated with *Aureobasidium pullulans* and held over an eight-week period to assess their ability to resist colonization. Figure 4 presents the fungal growth evolution during this eight-week period for samples coated with different formulations. Pictures of the coated samples before inoculation plus 150 h of accelerated UV exposure and after eight weeks of testing are presented in Figure 5. After two weeks of incubation, samples coated with the formulation without fungicides (formulation A) already showed staining at the growth rings and streaks of black stain across a few samples. Samples coated with the other formulations present no to small growth spots on the surface of the coatings. After four weeks, fungal growth is present on more than 50% of the surface of samples coated with formulation A. Very little black-stain growth is observed on samples coated with the reference formulation (formulation containing commercial fungicide (B)) and on samples coated with the formulation containing AgNPs prepared with AIBN (formulation D). No black-stain growth is observed on samples coated with the formulation containing AgNPs prepared with KPS (formulation C). After eight weeks, fungal growth is observed on all the samples.

The photographs in Figure 5 show that fungal growth obscures more than 70% of the surface of group A samples. Samples coated with formulation D present fungal growth similar to that observed for the reference formulation (B). Samples coated with formulation C present small streaks and spots of black stain, yet the overall fungal growth is less than that observed for samples coated with the reference formulation (B). It is important to note that this result was achieved with only 0.1 % (on total formulation solid weight) of AgNPs.

## 4. Discussion

The first notable difference observed between the latexes prepared with the two initiators is the efficiency of AgNPs incorporation. As reported above, ICP-OES analysis shows that there are approximately twice as many AgNPs embedded in the final dry film when AIBN is used as compared with KPS. This important observation is probably the result of differences in the nucleation step of the polymerization. Since KPS decomposes in the aqueous phase, it favors homogenous/micellar nucleation rather than nucleation in monomer droplets [21]. Latex particles nucleated in the aqueous phase will not contain AgNPs since the particles are stabilized with a hydrophobic ligand and initially dispersed within the monomer droplets. This observation differs from the results reported by Mori et al. [20] where polymerization using KPS was found to lead to efficient entrapment of hydrophobic magnetic particles in the polymer particles.

Although AgNP incorporation is more efficient in the case of the hydrophobic initiator, the ICP-OES results also indicate that not all the NPs are encapsulated. Various factors during the synthesis of the latexes can explain why the theoretical concentration of 10.00 mg/g was not obtained. Losses could occur as AgNPs are transferred from the vial to the beaker to the flask, through coagulum formation, and through nanoparticle sedimentation during the polymerization process.

Cryo-TEM was used to image the AgNPs distribution within the latexes particles. The differences between the two initiators appear to be related to the localization of the nanoparticles and their dispersion. The observation that the AgNPs are located closer to the surface of the acrylate particles and more evenly distributed when AIBN is used is in agreement with the results of Mori et al. [20] who reported that in the presence of a hydrophobic initiator, the metallic nanoparticles tend to be found closer to the surface due to the polymerization taking place preferentially inside the droplet and extending towards the outside of the droplets, pushing the metallic particles to the periphery of the particles. Moreover, the AIBN prepared latex shows relatively more monodisperse polymer particles than KPS-prepared latexes. Similar results were observed by Desbiens [27] in the synthesis of polystyrene latexes containing AgNPs with AIBN.

The dispersion of AgNPs in acrylic films was also examined using SEM. According to SEM analysis, the AgNPs encapsulated using the oil-soluble initiator present a relatively uniform dispersion in the free film. On the other hand, nanoparticles encapsulated in the water-soluble initiator tend to form larger aggregates. This is probably explained by the less uniform dispersion of AgNPs in the latex when using KPS (as seen with the cryo-TEM analysis on Figure 1), which is amplified following the coalescence of the polymer particles during film formation.

The difference between the antifungal properties of latexes containing AgNPs prepared with KPS as compared with those prepared with AIBN can provide some insight into the mechanism of the antifungal action of AgNPs. It is well known in the literature that the antifungal/antibacterial properties of AgNPs are influenced by various factors, including size [28], shape [29], stabilizing agent [30], and dispersion. However, the exact antibacterial mechanism of action of AgNPs is not entirely clear, and several contradictory studies on the actual mechanism of action of AgNPs are present in the literature. Wong and Liu proposed that due to their large surface area, AgNPs can attach to the cellular membrane and easily penetrate the cells. Once inside the cell, AgNPs can reach the cytoplasm and interact with sulfur/phosphorus-containing elements such as DNA [31,32]. Another study has demonstrated that AgNPs cause cell death through oxidative damage but not due to DNA modification [33]. There are also several discussions on the role of silver ions (Ag^+^) in the antimicrobial activity of AgNPs. Some authors [34,35] suggested that the mechanism of action of AgNPs is only through the release of Ag^+^ and that the importance of their large surface area in antimicrobial activity is negligible. According to Duran et al. [36], Ag^+^ ions react with the thiol groups of cell proteins, leading to their inactivation. Ag^+^ can also adhere to the cell wall due to its affinity for protein sulfhydryl groups. The permeability of the cytoplasmic membrane is consequently enhanced, thus deactivating respiratory enzymes, generating reactive oxygen species, and disrupting DNA replication [37]. On the other hand, proteomic analyses conducted by Lok et al. [38] established that AgNPs and Ag^+^ ions seem to have the same mechanism of action.

The results obtained in the current study corroborate the mechanism of action suggested by Ivask et al. [34] and Palza [35], where the antibacterial and antifungal properties are due to the release of Ag^+^ rather than the high surface area of the NPs. Indeed, the antifungal activity of AgNPs against *A. pullulans* seems to be higher when KPS is used as an initiator, even though the KPS-prepared latex has a lower AgNPs concentration. As shown by the SEM images, using KPS as an initiator leads to larger aggregates, which could generate higher local concentrations of Ag^+^. Similar conclusions were drawn by Khaydarov et al. [39], who report pronounced antibacterial/antifungal efficacy in AgNPs-embedded paints, even though the AgNPs tend to be agglomerated.

## 5. Conclusions

The nature of the initiator influences the incorporation and distribution of AgNPs in polymer particles synthesized by miniemulsion polymerization. AgNPs seem to be closer to the surface of the polymer particles and more evenly distributed when AIBN, a hydrophobic initiator, is used. One of our initial hypotheses was that a more even distribution of AgNPs would increase their antifungal properties. However, it seems to be quite the contrary: the antifungal efficiency of latexes containing AgNPs against *A. pullulans* is higher when KPS is used, despite this initiator leading to a less uniform dispersion of the nanoparticles and bigger aggregates. This suggests that *A. pullulans* is more affected by the concentration of Ag^+^ than by a uniform dispersion of smaller nanoparticles. Moreover, the relatively small concentration in AgNPs (0.1% on total solid weight) as a fungicide has proven to be as efficient as IPBC and propiconazole against *A. pullulans* in acrylic films.

## Figures and Tables

**Table 1:** Formulations prepared from latexes containing AgNPs mixed with a commercial acrylic resin, their identification, and fungicide concentrations.

Formulation	Description	Initiator	Theoretical Fungicide Concentration [% on Total Formulation Weight]	Experimental Fungicide Concentration [% on Total Formulation Weight] *	Experimental Fungicide Concentration [% on Total Solid Weight]
A	Latex without fungicide	KPS	-	-	-
B	Latex with IPBC and propiconazole	KPS	0.1 IPBC + 1 propiconazole	-	0.3 IPBC + 3 propiconazole
C	Latex with Ag NPs	KPS	0.11	0.04	0.1
D	Latex with Ag NPs	AIBN	0.11	0.06	0.2

* Concentration determined by ICP-OES.

**Table 2:** Parameters used for accelerated UV exposure.

Parameters	
Exposition Time	180 h
Filter	Daylight—Q
Irradiance (W/m^2^/nm)	0.35
Wavelength (nm)	340
Step 1	102 min of light, 30% RH, 63 °C (black panel temperature)
Exposition time	180 h

**Table 3:** Rating as a Function of Degree of Fungal Growth (Adapted from ASTM D5590-10).

Rating	Degree of Fungal Growth
0	No visible growth
1	Black stain covering up to 10% of surfaces providing growth is not as intense or colored as to obscure the sample color over more than 5% of surfaces.
2	Black stain covering between 10% and 30% of surfaces providing growth is not as intense or colored as to obscure the sample color on more than 10% of surfaces.
3	Black stain covering between 30% and 70% of surfaces providing growth is not as intense or colored as to obscure the sample color on more than 30% of surfaces.
4	Black stain on greater than 70% of surfaces providing growth is not as intense or colored as to obscure the sample color over more than 70% of surfaces.
5	Black stain on 100% of surfaces or with less than 100% coverage and with intense or colored growth obscuring greater than 70% of the sample color.

**Table 4:** AgNPs concentration in dry films determined by ICP-OES.

Formulations	Ag Concentration (mg/g) in Dry Film
With KPS	4.85
With AIBN	8.49

## Data Availability

Not applicable.
